# The involvement of human breastmilk-derived extracellular vesicles in the development of infant atopic sensitization 

**DOI:** 10.5414/ALX02651E

**Published:** 2026-09-10

**Authors:** Tinevimbo Weidner Baricholo, Amiq Gazdhar, Matthias V. Kopp, Emilie Seydoux

**Affiliations:** 1 Lung Precision Medicine (LPM), Department for Biomedical Research (DBMR),; 2 Division of Pediatric Respiratory Medicine and Allergology, Department of Pediatrics, and; 3 Department for Pulmonary Medicine, Allergology and Clinical Immunology, Inselspital, Bern University Hospital, University of Bern, Bern, Switzerland; *Both authors share last authorship.

**Keywords:** breastfeeding, breastmilk, extracellular vesicles, allergies, asthma, atopic sensitization

## Abstract

Breastmilk, with its unique biological components, is the optimal source of neonatal nutrition, and WHO global feeding guidance recommends that all infants are exclusively breastfed for the first 6 months. The composition of human milk depends on multiple factors such as maternal diet, exposure to pathogens, metabolic or autoimmune disorders, smoking, mental health, and genetics. Breastfeeding not only transfers nutrients to the child, but it also influences the infant immune function, through the transfer of immune cells, antibodies, microbiota from the mother, cytokines, or growth factors. Among these, human milk-derived extracellular vesicles (hMEVs) are emerging as key yet understudied elements in neonatal growth, development, and immunity. This review explores their structure, formation, uptake, and roles in supporting development, modulating immune responses during early life, and influencing allergic outcomes, highlighting their potential significance in infant health.

## Introduction 

Breastfeeding provides extensive short- and long-term health benefits for both children and mothers. Numerous studies consistently demonstrate that breastfed infants experience lower rates of illness and mortality compared with those who are formula-fed [1, 2]. For mothers, breastfeeding is linked to prolonged postpartum amenorrhea and a reduced risk of breast and ovarian cancers and type 2 diabetes [3], underscoring its vital role in maternal health. 

Despite the well-documented general benefits of breastfeeding, its impact on the development of allergies and asthma remains uncertain. While breastfeeding is often considered protective, results from systematic reviews and cohort studies have been inconsistent [4]. For instance, a New Zealand birth cohort found that breastfeeding for 4 weeks or longer increased the risk of allergen sensitization and asthma during mid-childhood [5]. Similarly, the Tucson Children’s Respiratory Study reported an elevated asthma risk in breastfed children of asthmatic mothers [6]. In contrast, the Tasmanian Longitudinal Health Study showed that exclusive breastfeeding was protective at age 7 but associated with higher asthma prevalence later in life [7]. Studies have shown that maternal general health may alter breastmilk composition and influence the child’s immune system [8, 9]; these findings further suggest that maternal asthma may exert similar effects. 

The early postnatal period represents a critical window for immune programming and the prevention of allergic diseases [10]. Breastmilk, as one of the first environmental exposures, may shape the trajectory of immune and allergic responses through its complex and dynamic composition. Human milk is ~ 87% water, 1% protein, 4% lipids, and 7% carbohydrates, along with a wide array of bioactive molecules such as vitamins, minerals, antibodies, immune cells, cytokines, and microbiota. These components collectively influence infant immune function and gut microbial colonization [11]. 

Among the numerous bioactive elements unique to breastmilk are human milk-derived extracellular vesicles (hMEVs). These nanosized vesicles, absent from infant formula, are increasingly recognized as important mediators of intercellular communication and molecular transport [12, 13]. Emerging evidence suggests that hMEVs play crucial roles in neonatal growth and immune development, yet their exact physiological functions in the context of asthma and allergies remain to be fully elucidated. Of importance, differences in the molecular cargo of hMEVs between healthy and asthmatic mothers may contribute to the varied effects of breastfeeding observed across studies. 

## Characteristics of hMEVs 

Extracellular vesicles (EVs) are lipid bilayer-enclosed structures secreted by nearly all cells in a variety of fluids, such as serum, urine, amniotic fluid, cerebrospinal fluid, and breastmilk [14]. Their cargo, comprised of proteins, lipids, and nucleic acids (DNA, mRNA, microRNA (miRNA)), is transferred between cells, appointing EVs as key mediators of intercellular communication. Importantly, the cargo largely depends on the origin and physiological state of the parent cell [15]. In breastmilk, hMEVs represent a heterogeneous population of exosomes (30 – 150 nm in size) and microvesicles (100 – 500 nm) [16] released not only by mammary epithelial cells but also by immune and stem cells from the breast and other organs [12]. 

Internalization into target cells through endocytosis, membrane fusion, or receptor-mediated pathways is enabled by the lipid bilayer membrane of hMEVs. Particularly, glycoproteins with sialic acid and other high-mannose and complex glycans were shown to influence how hMEVs interact with the infant’s cells [17]. hMEVs first interact with the infant’s oral cavity upon feeding, where they were shown to maintain the epithelial barrier integrity by accelerating re-epithelization of gingival epithelial cells upon injury [18]. hMEVs proceed to the gastrointestinal epithelial tissue, where studies have showed that the bilipid membrane of hMEVs can withstand the stringent acidic conditions in the stomach, thus protecting their cargo for potential absorption and subsequent biological functions in the infant gut [18, 19]. Neonatal human enteroids, organoids derived from intestinal tissue and composed solely of epithelial cells, were demonstrated to rapidly take up hMEVs by clathrin-mediated endocytosis [19]. 

Emerging evidence suggests that hMEVs may also reach the infant’s airways through aerosol inhalation during breastfeeding, potentially modulating inflammatory responses [20]. Following ingestion or inhalation, hMEVs can cross epithelial barriers into the circulation and distribute systemically [21]. Animal studies demonstrate that milk EVs can traverse the blood-brain barrier, notably accumulating in the hippocampus, cortex, and cerebellum [22]. Collectively, these findings highlight hMEVs as stable, bioactive carriers capable of systemic delivery of maternal molecular signals, which may influence infant immunity and development. 

## Physiological role of hMEVs on different organ systems 

Owing to their molecular heterogeneity, hMEVs influence diverse physiological processes across organ systems by activating specific signaling pathways. hMEVs were shown to enhance intestinal mucosal repair after injury by upregulating LGR5, a Wnt pathway co-receptor that drives stem cell differentiation into mesodermal and endodermal progenitors [23]. Maternal diet can further influence this process: a fiber-rich diet enriches hMEVs with miR-146a-5p, which activates Wnt signaling and promotes intestinal cell proliferation and migration in offspring [24]. hMEVs also strengthen epithelial barrier integrity by activating p38 MAPK- and MEK-ERK-dependent repair pathways and interacting with PI3K signaling, thereby supporting gut maturation [18]. 

Beyond the gut, hMEVs contribute to the development and function of other organ systems. In rodent models, milk-derived EVs accumulated in the brain and elicited distinct gene expression profiles that promote neuronal maturation, spatial learning, memory, and seizure resistance [22]. hMEVs also enhanced skeletal muscle growth by activating PI3K and AKT-mTOR-p70s6k pathways, increasing myotube diameter, muscle fiber density, and exercise performance [25]. In bone tissue, orally administered milk EVs increased osteocyte numbers, enhanced proliferation, upregulated osteogenic gene expression, and improved bone repair capacity [26]. 

## Immunomodulatory potential of hMEVs on infant immunity 

Because a portion of hMEVs originate from immune cells, they carry unique immune-related cargo that influences oral and gastrointestinal immunity [27]. In experimental necrotizing enterocolitis (NEC), EVs from colostrum, transitional, and mature milk protected against inflammatory injury, with colostrum-derived EVs being most effective [28]. Both intraperitoneal and oral delivery of milk EVs reduced NEC incidence and severity, partly by decreasing oxidative stress and epithelial cell death [29, 30]. Human milk EVs also inhibited agonist-induced endosomal TLR3 and TLR9 signaling in gingival epithelial cells, lowering IL-6 and CXCL10 expression, and they helped maintain CD4^+^ T cells in a naïve state [18]. Functional differences also emerge with gestational age: term hMEVs suppressed IL-6 secretion in intestinal epithelial cells and reduced IL-1β expression in macrophages more effectively than preterm hMEVs [31]. 

Additional studies show broader immunomodulatory benefits. Bovine milk EVs delayed arthritis onset in mouse models, reduced cartilage pathology, and lowered levels of MCP-1, IL-6, and anti-collagen IgG2a [32]. Human milk EVs inhibited HIV-1 infection of dendritic cells and prevented viral transfer to CD4^+^ T cells [33], as well as prevented infection by rotaviruses, respiratory syncytial viruses and human cytomegaloviruses [34, 35]. They may also protect the infant respiratory tract: hMEVs preserved epithelial barrier integrity in vitro and reduced polyI:C-induced TNF-α, IL-8, and IL-6 production in a bronchial epithelial cell line [20]. 

Much research on hMEV cargo has focused on miRNAs, the most abundant intravesicular component. miRNAs regulate gene expression post-transcriptionally and play key roles in development and immune function [36]. Maternal conditions influence miRNA composition: obese mothers show upregulation of several neurologically relevant miRNAs [37], and large cohort studies have identified clusters linked to endocrine and neurodevelopmental pathways [38]. In type 1 diabetes, 9 miRNAs in hMEVs are aberrantly expressed, although their effects on infant immunity remain unclear [39]. Other immune-modulatory miRNAs found to be transported by hMEVs are summarized in Table 1. 

## hMEVs and infant atopic sensitization 

Atopic sensitization reflects a predisposition to exaggerated immune responses due to exposure to specific environmental or dietary antigens. Understanding whether hMEVs contained in breastmilk and transferred during breastfeeding have an impact on the development of allergies is of central importance, particularly given the rising global prevalence of pediatric asthma, allergic rhinoconjunctivitis, food allergies, and eczema in pediatric populations [47, 48]. No dedicated studies have directly investigated a potential relationship between EVs, breast milk, and atopic sensitization in infants, highlighting a substantial gap in current knowledge. Nevertheless, indirect evidence from related lines of research suggests a possible association. 

In asthma, the expression of specific miRNA (miR-221 and miR-221-3p) was significantly increased in bronchial epithelial cells and sputum, respectively, from asthmatic adult subjects [49, 50]. Whether these are found in breastmilk of asthmatic mothers is unknown. Bozack et al. [51] identified in hMEVs nine distinct miRNAs associated with maternal asthma during pregnancy (e.g., miR-324-5p, miR-106b-5p), with miR-1290 linked to both asthma and atopy. Whether these have an impact on infant health remains to be elucidated. Another study reported that airway epithelial cells release pro-inflammatory EVs in response to rhinovirus infection, which is a major driver of early-childhood asthma exacerbations [52]. Conversely, some hMEVs may exert tolerogenic effects. Exosomes enriched with miR-155 were shown to modulate the IL-4/Th2 axis and regulate FoxP3 and FcεRI expression, suggesting potential roles in suppressing atopic sensitization [53]. 

Understanding of the relationship between food allergies and hMEVs also remains in its early stages. Recent data show that infants who received partial colostrum feeding had a higher risk of developing peanut allergy at 12 – 18 months compared with infants who received exclusive colostrum feeding [54]. Although the study does not directly investigate hMEVs, its observation that colostrum intake is associated with protection aligns with other research suggesting that colostrum, rich in hMEVs, may confer specific benefits. Profiling of miRNA cargo in serum EVs from children with food-induced anaphylaxis revealed that miR-21-3p and miR-487b-3p, both associated with inflammation- and immune regulation-related pathways, were elevated during acute episodes compared with baseline levels. [55]. EVs isolated from monocyte-derived dendritic cells of children with IgE-mediated cow’s milk allergy, but not from healthy children, promoted increased proliferation of Th2 cells, along with enhanced eosinophil ROS production and migration [56]. These findings highlight the pro-inflammatory potential of EVs in allergic contexts, but it remains unclear whether such EVs are present in breastmilk or influence the development of allergies in infants. 

Overall, hMEVs remain insufficiently characterized, and additional research into their cargo and immunomodulatory properties (Table 2) is essential to determine whether they support immune tolerance or contribute to sensitization in early life. Understanding the mechanisms by which hMEVs influence early immune programming could ultimately impact breastfeeding guidelines, particularly in settings involving maternal allergic disease. Careful documentation of maternal allergy status before, during, and after pregnancy as well as early-stage analysis of hMEVs composition may prove valuable for identifying potential risk factors without prematurely suggesting changes to breastfeeding practices. In addition, the use of hMEVs as targeted delivery vehicles opens new avenues for the development of advanced immune-modulatory therapeutics. These could be engineered to modulate immune responses implicated in the onset of allergic diseases, and new infant formulas could incorporate hMEVs targeted at specific cell populations. Such advances have the potential to contribute to personalized strategies aimed at reducing the growing global burden of allergies and asthma in children. 

## Authors’ contributions 

Tinevimbo Weidner Baricholo: Conception of review, literature search, draft of review. Amiq Gazdhar: Critical evaluation of intellectual content. Matthias V. Kopp: Draft of review, critical evaluation of intellectual content, financial support. Shares last authorship. Emilie Seydoux: Conception of review, literature search, draft of review, critical evaluation of intellectual content. Shares last authorship. 

## Funding 

This review was supported by the Department of Pediatrics, Inselspital, Bern University Hospital, Bern, Switzerland. 

## Conflict of interest 

The authors declare no conflict of interest. 

**Table 1.** Examples of microRNA isolated from human breastmilk, which have important function in immune processes modulation.

**miRNA**	**Function**	**References**
miR-148	Regulation of immune response, inflammation regulation, modulation of intestinal epithelial cells	[40]
miR-181	Regulates the differentiation of B cells and CD4^+^ cells	[41]
miR-17	Regulates the development of B cells, T cells and monocytes	[41]
miR-223	Associated with the proliferation of granulocytes	[42]
miR-30-5b-5b	Associated with promoting cellular invasion and immunosuppression	[43]
miR-182-5p	Promotes T cell-mediated immune responses	[43]
miRNA-148a-3p	Associated with the regulation of the processes of maturation and differentiation of B and T cells	[44]
miRNA-155	Differentiation of B and T cells, IL-4 signaling, immunoglobulin class switching to FcRI expression	[44]
miRNA-99b-5p	Differentiation and regulation of B and T cells	[44]
miRNA-16-5p		
miRNA-182-5p		
miRNA-181a-5p		
miR-155	IL-4 signaling, immunoglobulin class switching to IgE and FcεRI expression Associated with mild asthma and targets the IL-13 pathway	[45, 46]

**Table 2.** Potential lines of research in the field of human milk-derived extracellular vesicles and their relation to allergic diseases.

1. Standardization of hMEV isolation and characterization methods;
2. Longitudinal studies linking maternal health to hMEV profiles;
3. Impact of environmental and lifestyle factors on hMEV composition;
4. Mechanistic studies on hMEV-mediated immune modulation;
5. Integration of multi-omics approaches to study hMEV cargo;
6. Maternal allergy screening and early hMEV profiling;
7. Exploration of hMEVs as therapeutic or preventive tools
